# Glioma-Specific Diffusion Signature in Diffusion Kurtosis Imaging

**DOI:** 10.3390/jcm10112325

**Published:** 2021-05-26

**Authors:** Johann-Martin Hempel, Cornelia Brendle, Sasan Darius Adib, Felix Behling, Ghazaleh Tabatabai, Salvador Castaneda Vega, Jens Schittenhelm, Ulrike Ernemann, Uwe Klose

**Affiliations:** 1 Department of Neuroradiology, University Hospital Tübingen, Eberhard Karls University, 72076 Tübingen, Germany; cornelia.brendle@med.uni-tuebingen.de (C.B.); ulrike.ernemann@med.uni-tuebingen.de (U.E.); uwe.klose@med.uni-tuebingen.de (U.K.); 2 Center for CNS Tumors, Comprehensive Cancer Center Tübingen—Stuttgart, University Hospital Tübingen, Eberhard Karls University, 72076 Tübingen, Germany; sasan.adib@med.uni-tuebingen.de (S.D.A.); felix.behling@med.uni-tuebingen.de (F.B.); ghazaleh.tabatabai@med.uni-tuebingen.de (G.T.); salvador.castaneda@med.uni-tuebingen.de (S.C.V.); jens.schittenhelm@med.uni-tuebingen.de (J.S.); 3 Department of Neurosurgery, University Hospital Tübingen, Eberhard Karls University, 72076 Tübingen, Germany; 4 Departments of Neurology and Neurosurgery, Interdisciplinary Division of Neuro-Oncology, University Hospital Tübingen, Hertie Institute for Clinical Brain Research, Eberhard Karls University, 72076 Tübingen, Germany; 5 German Cancer Consortium (DKTK), DKFZ Partner Site Tübingen, 72076 Tübingen, Germany; 6 Department of Pathology and Neuropathology, Institute of Neuropathology, University Hospital Tübingen, Eberhard Karls University, 72076 Tübingen, Germany; 7 Werner Siemens Imaging Center, Department of Preclinical Imaging and Radiopharmacy, University Hospital Tübingen, Eberhard Karls University, 72076 Tübingen, Germany

**Keywords:** glioma, diffusion kurtosis imaging, mean kurtosis, mean diffusivity, diffusion signature, segmentation

## Abstract

Purpose: This study aimed to assess the relationship between mean kurtosis (MK) and mean diffusivity (MD) values from whole-brain diffusion kurtosis imaging (DKI) parametric maps in preoperative magnetic resonance (MR) images from 2016 World Health Organization Classification of Tumors of the Central Nervous System integrated glioma groups. Methods: Seventy-seven patients with histopathologically confirmed treatment-naïve glioma were retrospectively assessed between 1 August 2013 and 30 October 2017. The area on scatter plots with a specific combination of MK and MD values, not occurring in the healthy brain, was labeled, and the corresponding voxels were visualized on the fluid-attenuated inversion recovery (FLAIR) images. Reversely, the labeled voxels were compared to those of the manually segmented tumor volume, and the Dice similarity coefficient was used to investigate their spatial overlap. Results: A specific combination of MK and MD values in whole-brain DKI maps, visualized on a two-dimensional scatter plot, exclusively occurs in glioma tissue including the perifocal infiltrative zone and is absent in tissue of the normal brain or from other intracranial compartments. Conclusions: A unique diffusion signature with a specific combination of MK and MD values from whole-brain DKI can identify diffuse glioma without any previous segmentation. This feature might influence artificial intelligence algorithms for automatic tumor segmentation and provide new aspects of tumor heterogeneity.

## 1. Introduction

Gliomas are the most frequent primary tumors of the central nervous system. They have a global incidence of 0.006% [1], a poor prognosis, and high morbidity [2]. The revised 4th edition of the World Health Organization classification of tumors of the central nervous system (2016 CNS WHO) combines both histological and molecular characteristics into an “integrated diagnosis” [3]. Molecular stratification is fundamental to estimate individual prognosis [4,5,6]. The relevant molecular markers comprise the isocitrate-dehydrogenase (IDH) 1/2 mutation status and co-deletion of chromosome 1p/19q (loss of heterozygosity; LOH). Both the co-deletion of chromosome 1p/19q and the IDH1/2 mutation are favorable prognostic factors in gliomas [4,5,6,7,8,9,10]. As a complementary molecular marker, alpha-thalassemia/mental retardation syndrome X-linked (ATRX) is predictive for associated hotspot mutations of IDH or H3 histone, family 3A (H3F3A) [11]. The loss of ATRX expression results in a phenotype characterized by alternative lengthening of telomeres (ALT) [12]. This [9] as well as the O6-methylguanine DNA methyltransferase (MGMT) methylation status [13,14,15] are also a favorable prognostic factor in glioma.

Treatment planning, intraoperative navigation, and monitoring of potential tumor recurrence during or after treatment rely on advanced magnetic resonance imaging (MRI) [16,17] methods. Specifically, tumor segmentation allows for volumetric analysis, which is the basis for the planning of radiotherapy [18], treatment monitoring [19,20], and the calculation of the extent of glioma resection [21]. Manual tumor delineation by experienced radiologists is currently the gold standard for tumor segmentation, despite requiring high expenditure of time and presenting technical challenges [20,22,23] and potentially low inter-reader agreement in postoperative MR examinations or in evaluating non-enhancing tumor parts [17,24]. However, automatic tumor segmentation is an emerging field [25].

The apparent kurtosis coefficient (AKC) is a dimensionless metric from diffusion kurtosis imaging (DKI). The AKC quantifies the degree of deviation from the Gaussian distribution of the diffusion-induced signal decay [26,27,28]. DKI has evolved from the diffusion-weighted imaging (DWI) method and uses multiple and high b-values [26,27,28,29,30,31]. The essential DKI metrics are the mean kurtosis (MK) and the mean diffusivity (MD) [28,31]. They enable a quantitative assessment of water diffusion behavior in biological tissues such as the brain. However, diffusion barriers alter the water diffusion probability distribution. Thus, DKI may be a surrogate parameter for a tissue’s microstructural composition, complexity, and heterogeneity [27,29]. Previously, DKI has shown potential in distinguishing between histopathological and molecular glioma features [32,33] or as a prognostic factor in diffuse glioma [34].

In their initial publication introducing the DKI technique, Jensen et al. first demonstrated the correlation between MK and MD values from whole-brain DKI parametric maps in healthy subjects on a scatter plot [26]. However, we discovered a specific and until then undescribed distribution pattern of MK and MD values in glioma patients from a previous cohort [34]. Therefore, this study sought to explore this phenomenon and its potential implications for automatic tumor segmentation.

## 2. Materials and Methods

### 2.1. Study Design and Ethics

This retrospective cross-sectional observational analysis complies with the STROBE guidelines [35] and was conducted on the principles of the “International Conference on Harmonization: Good Clinical Practice guidelines” and the latest version of the Declaration of Helsinki. The local institutional review board approved this study (Ref. No. 727/2017BO2) and waived the written informed consent due to the retrospective study design.

### 2.2. Patient Selection and Stratification

**Figure 1:**
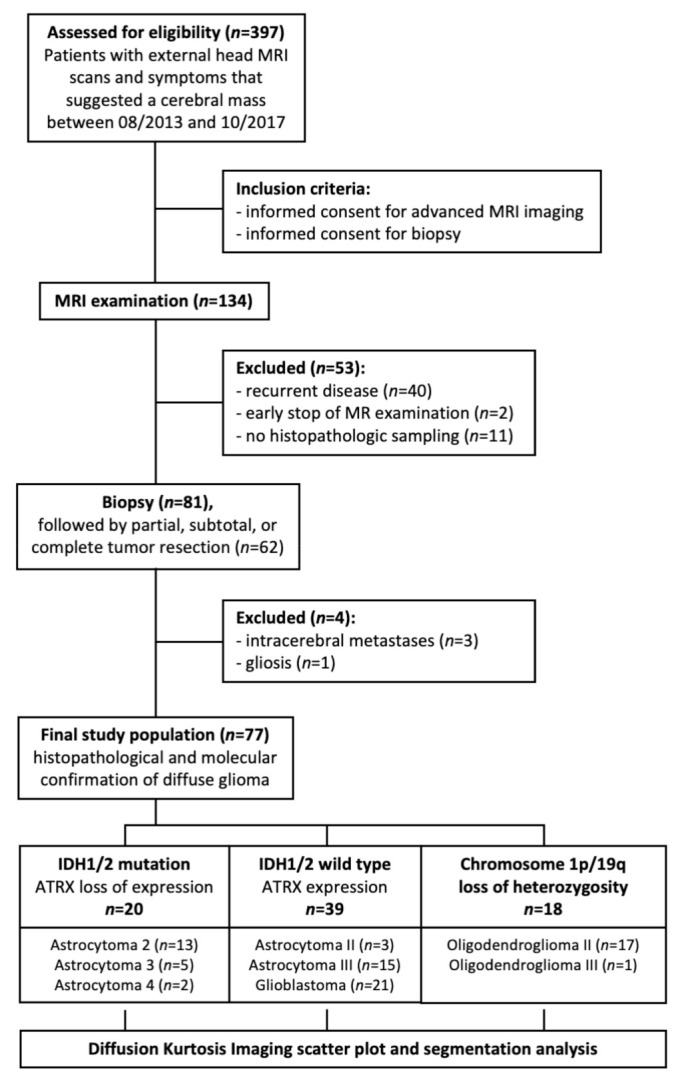
Patient flow diagram according to the STROBE guidelines. MRI, magnetic resonance imaging; IDH, isocitrate-dehydrogenase.

As previously described, we selected the study cohort from 397 consecutive patients with diffuse glioma diagnoses from 1 August 2013 and 30 October 2017 [34]. Figure 1 shows the patient selection and dichotomization algorithm using a flow diagram.

The final study group encompassed 77 patients and consisted of 43 men (56%) and 34 women (44%). The mean age was 53.1 ± 15.7 years. In the control group, we retrospectively selected seven healthy volunteer subjects and one from another study cohort comprising four males and three females with a mean age of 31.2 ± 12.2.

Glioma grading is based on histopathological examinations and full immunohistochemical workup of specimens obtained from partial, subtotal (>90%), or complete tumor resection in patients [34]. Including histopathological and molecular data, the glioma classification complies with the current 2016 CNS WHO criteria [3]. In the integrated approach, the combination of loss of ATRX expression and presence of IDH1/2 mutation characterized IDH_mut_ astrocytoma (AS), including its most aggressive histological subtype of astrocytoma, IDH-mutant, WHO grade 4 according to the cIMPACT-NOW update 5 [36]. Tumors with a wild-type IDH (IDH_WT_) status and retaining ATRX expression are primary glioblastomas (GBMs). Oligodendrogliomas were defined by the synchronous co-deletion of chromosome 1p/19q and IDH1/2 mutation, whereas an overwhelming majority maintain ATRX expression [1,7,30]. Additionally, IDH_mut_ diffuse AS WHO grade 2 (AS2), IDH_mut_ anaplastic AS WHO grade 3 (AS3), and IDH_mut_ AS WHO grade 4 (AS4) were grouped as (1) IDH_mut_ AS; IDH_wt_ AS2, IDH_wt_ AS3, and IDH_wt_ AS4/GBM were grouped as (2) IDH_wt_ GBM, and 1p/19q-confirmed diffuse (OD2) and anaplastic oligodendrogliomas (OD3) were grouped as (3) OD_1p/19q-LOH_, based on their integrated molecular profiles as well as their clinical outcomes [4,5,9,10].

### 2.3. Procedures and Techniques

#### 2.3.1. MR Imaging

As previously reported [34], MR imaging used a 3.0 T MRI scanner (Biograph mMR, Siemens Healthcare, Erlangen, Germany) with a 32-channel head coil. The conventional MR examination protocol included a transversal 2D-encoded T2-weighted fluid-attenuated inversion recovery (FLAIR) sequence (TR/TE, 9000/87 ms; inversion time (TI), 2500 ms; slice number, 40; slice thickness, 3 mm) and a sagittal 3D-encoded isotropic magnetization prepared rapid acquisition gradient echo (MPRAGE) sequence (TR/TE, 1900/2.4 ms; TI, 900 ms; slice number, 124; slice thickness, 1.0 mm) before and after administration of 0.1 mL/kg body weight gadobutrol (Gadovist^®^, Bayer, Leverkusen, Germany). DKI used a spin-echo 2D echo-planar imaging DWI sequence with implemented b-values of 0, 500, 1000, 1500, 2000, 2500 s/mm^2^, and diffusion encoding in 30 directions for each of these values. The other imaging parameters were as follows: TR, 5900 ms; TE, 95 ms; matrix, 128 × 128; slice thickness, 5 mm; slice number, 25; field of view, 250 × 250 mm^2^; bandwidth, 965 Hz/pixel; parallel imaging with a sensitivity encoding factor of 2 in the anteroposterior direction.

#### 2.3.2. Image Post-Processing and Analysis

As previously described [32], the MD and MK parametric maps were calculated after precedent smoothing using the MR Body Diffusion tool^®^ V.1.4.0 in syngo.via frontier^®^ (Siemens Healthcare, Erlangen, Germany). Image and volume of interest (VOI) analyses were performed on the parametric maps using MIPAV 10.0.0 (http://mipav.cit.nih.gov; access date 1 May 2021). The entire tumor volume was manually delineated on multiple slices on the FLAIR images, as indicated by T2 signal alterations. We minimized potential sampling bias by encompassing T2 hyperintense areas showing peritumoral edema and perifocal infiltrative zone [37,38]. Then, we transformed the MD and MK parametric maps on the transverse FLAIR-weighted images’ matrix using in-house Matlab-based algorithms (Matlab 2018b, MathWorks, Natick, MA, USA). Subsequently, we extracted the MK and MD intensity values voxel-wisely from the whole-brain MD and MK parametric maps and processed them for further analysis using Matlab (Matlab 2018b, MathWorks, Natick, MA, USA).

### 2.4. Statistical Analyses

Data analyses used Matlab (Matlab 2018b, MathWorks, Natick, MA, USA) and IBM SPSS Statistics^®^ Version 27 (IBM, Armonk, NY, USA). The MK and MD values for integrated molecular glioma subgroups and healthy control groups were displayed on scatter plots. They were then compared to each other visually. We identified a tumor-specific combination of MK and MD values on the scatter plot in the glioma group, which does not occur in other brain or intracranial compartments and goes beyond the healthy brains’ MK and MD values of the control groups.

The corresponding voxels from the scatter plots were labeled and highlighted both on the DKI parametric maps and on the anatomic FLAIR images. Reversely, we compared the automatically labeled voxels with those of the manually segmented tumor volume, and the Dice similarity coefficient was then determined to investigate their spatial overlap.

## 3. Results

### 3.1. Distribution of MK and MD Values in Whole-Brain DKI Maps

**Figure 2:**
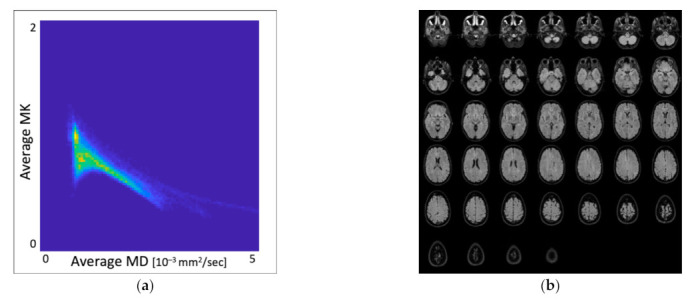
(**a**) Scatter plot with MK and MD values distribution in whole-brain DKI parametric maps in the healthy brain; MK is dimensionless; MD [10^−3^ mm^2^/s], (**b**) Corresponding batch of anatomical FLAIR images. MK, mean kurtosis; MD, mean diffusivity.

Figure 2 illustrates a scatter plot with MK and MD values distribution from whole-brain DKI parametric maps in the healthy brain.

**Figure 3:**
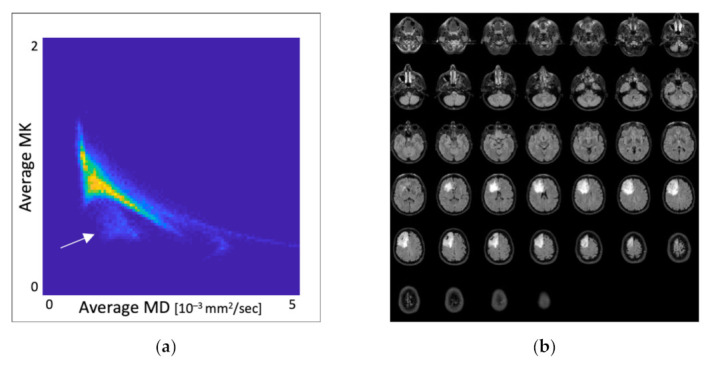
(**a**) Scatter plot with pathological MK and MD values distribution in whole-brain DKI parametric maps. The white arrow shows the separate voxels area with glioma-specific diffusion properties; MK is dimensionless; MD [10^−3^ mm^2^/s], (**b**) Corresponding batch of anatomical FLAIR images from a patient with chromosome 1p/19a co-deleted oligodendroglioma in the right frontal lobe.

Figure 3 demonstrates an anomalous voxel area with corresponding MK and MD values in whole-brain DKI parametric maps from an OD_1p/19q-LOH_ patient.

**Figure 4:**
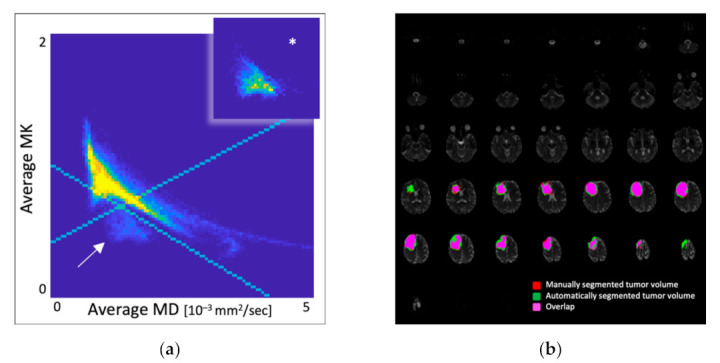
(**a**) Scatter plot with pathological MK and MD values distribution in whole-brain DKI parametric maps. The white arrow shows the separate voxels area with glioma-specific diffusion properties; the asterisked field shows the manual glioma segmentation’s voxel distribution; MK is dimensionless; MD [10^−3^ mm^2^/s], (**b**) Corresponding batch of MD images with the overlaid labels of diffusion-based automatic segmentation (green) and the manual segmentation (red) of the tumor and their spatial overlap (pink). Notably, the automatically labeled voxels do not occur in other brain or intracranial compartments.

Figure 4 shows that the labeled voxels from the MK and MD scatter plots are exclusively located in tumor tissue, regardless of the specific molecular glioma profile. Furthermore, they do not occur outside the area of the manually segmented tumor volume.

### 3.2. Overlap Analysis

Table 1 shows the degree of similarity between the automatically labeled voxels and the manually segmented tumor volume voxels among integrated glioma subgroups.

## 4. Discussion

This study aimed to explore the relationship between MK and MD values from whole-brain DKI parametric maps in 2016 CNS WHO integrated glioma groups. We identified a specific diffusion signature of diffuse glioma in whole-brain DKI characterized by a particular combination of MK and MD values, which does not occur in other brain or intracranial compartments.

In their initial publication presenting the technique of DKI, Jensen et al. demonstrated a scatter plot with MK and MD values of normal brain tissue [26]. After this, only quantitative MK and MD histogram analysis from DKI using manual, semi-automatic, or fully-automatic glioma segmentation has been applied to grade glioma [32,33,39,40,41,42], assess glioma heterogeneity [38,43,44], or perform a survival analysis [37]. Kickingereder et al. included DKI parameters into a machine-learning-based approach to identify molecular glioma characteristics [45]. Rulseh and Vymazal used quantitative ADC values from whole-brain DWI to assess overall and progression-free survival in GBM patients [46]. However, this is the first study since 2005 further evaluating the relationship between MK and MD values in whole-brain DKI maps from glioma patients.

The healthy brain shows a specific distribution of MK and MD values in whole-brain DKI maps on a scatter plot [26]. Our healthy control group’s findings correspond to those of Jensen et al. [26] showing similar MK or MD value distribution on the scatter plots and supporting our data’s validity.

In the 2016 CNS WHO-based glioma groups, our results show that diffuse glioma features a specific combination of MK and MD values in whole-brain DKI on scatter plots. Voxels with this diffusion signature do not occur in other brain or intracranial compartments. Moreover, these labeled voxels are almost exclusively located within the manually segmented tumor volume, as shown by the Dice similarity coefficient. This finding implies a potential way to automatically detect and segment glioma tissue on DKI parametric maps without any previous manual intervention. It might also influence artificial intelligence algorithms for automatic tumor segmentation and provide new aspects of tumor heterogeneity. In this regard, we did not find any corresponding reports in the literature. However, the labeled voxels harboring the tumor-specific diffusion value combination did not encompass the entire manually segmented tumor volume on anatomic FLAIR images but most areas within the tumor. We could not identify any systematics or specific distribution patterns from the visual impression. An influential factor may be the significant heterogeneity within the entity of glioma regarding morphology, microstructural properties, histopathological tumor grade, cell density, and cell or vessel proliferation [47,48,49], and the MK and MD in DKI maps may reflect these heterogeneities [27,29,38].

Additionally, there are automatically labeled voxels exceeding the manually segmented tumor VOI. A reason might be the perifocal infiltrative zone harboring increased cellularity or a higher amount of non-infiltrated brain tissue reflected by DKI, which may not show any pathological T2 signal alteration [47,48,49]. Small outlying parts of the automatically labeled voxels go beyond the manual VOI at the tumor margins. We found one potential explanation for this observation in the different slice thickness and inter-slice distance factor between DKI parametric maps and the anatomical FLAIR images, as we interpolated the DKI parametric maps to the anatomic FLAIR images and delineated the tumor volume on the FLAIR images, as outlined above (see Section 2.3.2 and Supplementary Figure S1). This divergence should be solved with a similar slice thickness and distance factor in future investigations.

Further investigation of this phenomenon is needed to correlate our findings with other anatomic or functional MRI measurements or metabolic imaging findings or evaluate them in post-treatment settings.

### Limitations

In this observational study, we did not assess the prevalence of glioma-specific diffusion properties in other intracranial pathologies such as inflammatory disease or other tumor entities, nor did we apply this method to glioma post-treatment monitoring. These aspects will be the subject of future investigations. Secondly, we did not perform VOI delineation on other sequences than the T2w/FLAIR, e.g., post-Gadolinium T1-weighted images. However, many of the lower-grade gliomas in our cohort did not show contrast enhancement. Excluding all non-enhancing lesions would automatically increase the selection bias. Thirdly, the process of VOI delineation may have been subject to sampling bias, because glioma infiltration may extend beyond T2 signal abnormalities [50,51]. However, studies have shown that the difference in tumor delineation among different observers has a minor impact because of the large number of voxels included in the histogram analysis [52,53]. Fourthly, we experienced a minor sampling bias regarding VOI overlap at the tumor margins because of the different slice thickness and distance factor between anatomical FLAIR images and DKI parametric maps. We will address this problem by harmonizing these parameters in future investigations. The final study limitation is the retrospective study design.

## 5. Conclusions

A unique diffusion signature with a specific combination of MK and MD values from whole-brain DKI can identify diffuse glioma without any previous segmentation, regardless of its molecular profile. Voxels with this diffusion signature do not appear in other brain or intracranial compartments. This feature might influence artificial intelligence algorithms for automatic tumor segmentation and provide new aspects of tumor heterogeneity.

## Figures and Tables

**Table 1:** Dice similarity coefficient for automatically labeled and manually segmented voxels among molecular glioma subgroups. IDH, isocitrate-dehydrogenase.

Molecular Glioma Group	Dice Coefficient
Astrocytoma, IDH1/2 mutation and loss of ATRX expression (*n* = 20)	0.79
Astrocytoma, IDH wild type and retained ATRX expression (*n* = 39)	0.73
OD_1p/19q-LOH_ (*n* = 18)	0.82
Average (*n* = 77)	0.78

## Data Availability

All data related to this study can be provided by the authors upon request.

## Supplementary Materials

The following are available online at https://www.mdpi.com/article/10.3390/jcm10112325/s1, Figure S1: VOI overlap between FLAIR images and DKI parametric maps in outlying tumor parts and infiltrative zone.
