# Supplementary material for: Glioma-Specific Diffusion Signature in Diffusion Kurtosis Imaging
Source: J Clin Med. 2021 May 26;10(11):2325. doi: 10.3390/jcm10112325 (PMC8199055; doi:10.3390/jcm10112325)
Supplement: Supplementary file 1 [file jcm-10-02325-s001.zip › jcm-1181345-supplementary.pdf]

Journal of Clinical Medicine

Supplementary Material

Glioma-Specific Diffusion Signature in Diffusion Kurtosis Imaging

Johann-Martin Hempel [1,2,*], Cornelia Brendle [1,2], Sasan Darius Adib [2,3], Felix Behling [2,3,4], Ghazaleh Tabatabai [2,4,5,7], Salvador Castaneda Vega [2,6], Jens Schittenhelm [2,7], Ulrike Ernemann [1,2] and Uwe Klose [1]

1 Department of Neuroradiology, University Hospital Tübingen, Eberhard Karls University, 72076 Tübingen, Germany; cornelia.brendle@med.uni-tuebingen.de (C.B.); ulrike.ernemann@med.uni-tuebingen.de (U.E.); uwe.klose@med.uni-tuebingen.de (U.K.)
2 Center for CNS Tumors, Comprehensive Cancer Center Tübingen—Stuttgart, University Hospital Tübingen, Eberhard Karls University, 72076 Tübingen, Germany; sasan.adib@med.uni-tuebingen.de (S.D.A.); felix.behling@med.uni-tuebingen.de (F.B.); ghazaleh.tabatabai@med.uni-tuebingen.de (G.T.); salvador.castaneda@med.uni-tuebingen.de (S. C. V.); jens.schittenhelm@med.uni-tuebingen.de (J.S.)
3 Department of Neurosurgery, University Hospital Tübingen, Eberhard Karls University, 72076 Tübingen, Germany
4 Interdisciplinary Division of Neuro-Oncology, Departments of Neurology and Neurosurgery, University Hospital Tübingen, Hertie Institute for Clinical Brain Research, Eberhard Karls University, 72076 Tübingen, Germany
5 German Cancer Consortium (DKTK), DKFZ partner site Tübingen, 72076 Tübingen, Germany
6 Werner Siemens Imaging Center, Department of Preclinical Imaging and Radiopharmacy, University Hospital Tübingen, Eberhard Karls University, 72076 Tübingen, Germany
7 Institute of Neuropathology, Department of Pathology and Neuropathology, University Hospital Tübingen, Eberhard Karls University, 72076 Tübingen, Germany
* Correspondence: johann-martin.hempel@uni-tuebingen.de

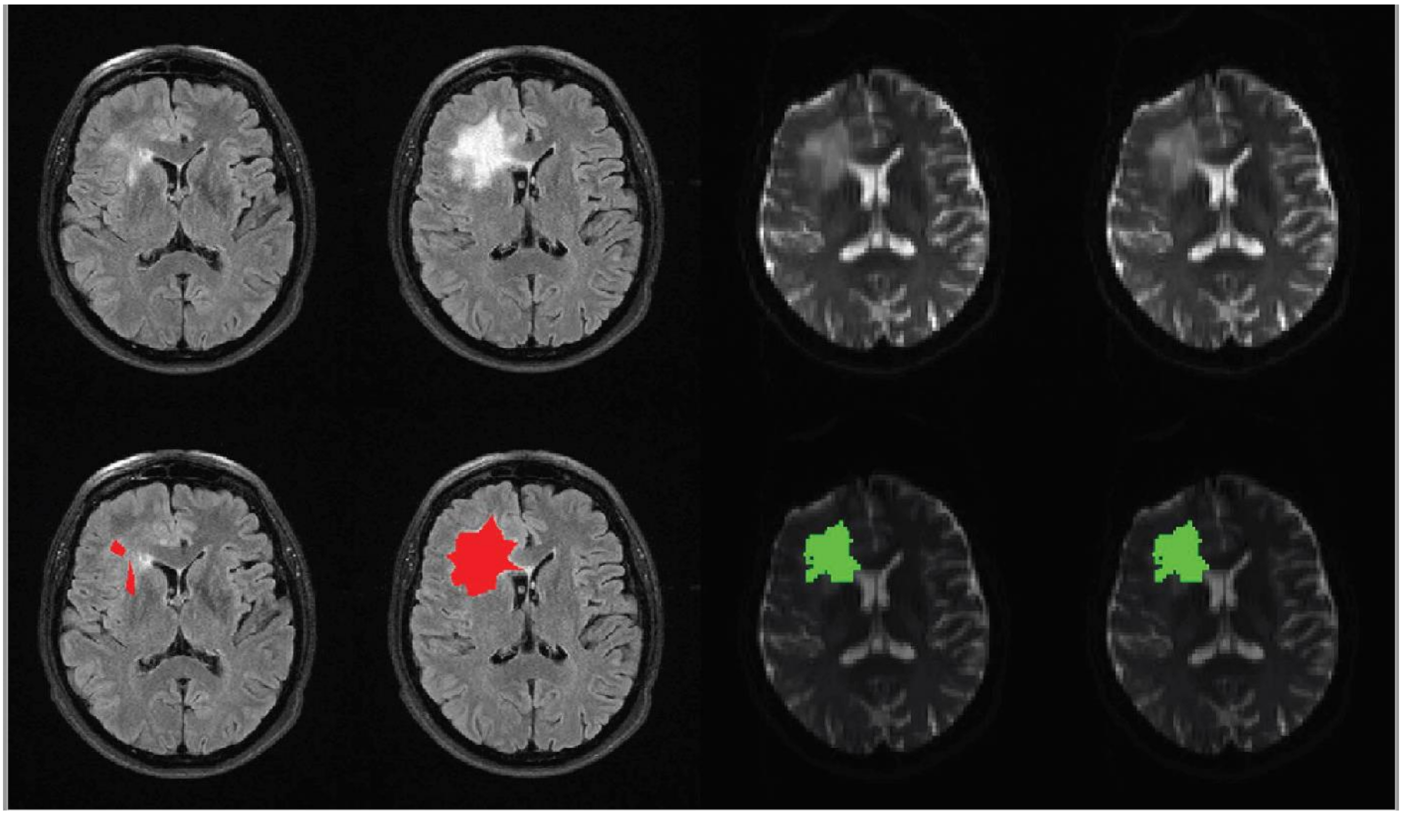

Figure 1. VOI overlap between FLAIR images and DKI parametric maps in outlying tumor parts and infiltrative zone.
